# Benign Neurofibroma/Schwannoma Hybrid Peripheral Nerve Sheath Tumor of the Ulnar Nerve Harboring a Metastatic Papillary Thyroid Carcinoma Deposit: A Case Report of Tumor-to-Tumor Metastasis

**DOI:** 10.1155/2022/9038222

**Published:** 2022-12-14

**Authors:** Juan M. Colazo, Alexander N. Perez, Anthony D. Judice, Julia Quirion, Carlos N. Prieto-Granada, Ginger E. Holt

**Affiliations:** ^1^Medical Scientist Training Program, Vanderbilt University School of Medicine, Nashville, TN, USA; ^2^Department of Pathology, Microbiology, and Immunology, Vanderbilt University Medical Center, Nashville, TN, USA; ^3^Department of Orthopaedic Surgery, Vanderbilt University Medical Center, Nashville, TN, USA

## Abstract

A 74-year-old man with a medical history significant for papillary thyroid cancer (PTC) presented with a rapidly enlarging grape-sized mass in his right medial arm with paresthesia in the ulnar nerve distribution. Imaging was suspicious for a peripheral nerve sheath tumor (PNST), but an ultrasound-guided biopsy was equivocal. The mass was excised with final histopathology demonstrating a benign neurofibroma/schwannoma hybrid nerve sheath tumor (N/S HNST) harboring a metastatic PTC deposit, ultimately mimicking the rare glandular schwannoma subtype. Next-generation sequencing (NGS) of the lesion demonstrated somatic variants in BRAF and TERT (common in PTC) and NF2 (common in PNSTs). After excision, the patient's nerve symptoms improved. A postsurgical PET/CT scan also showed progression in the lungs/mediastinum. Due to the metastatic nature of his PTC, he was treated with 14 mg of Lenvima (lenvatinib) daily, and his PET/CT surveillance was performed at more frequent intervals. Tumor-to-tumor metastasis (TTM) is a rare occurrence. To our knowledge, this is the first case reported on PTC metastasizing into a benign (hybrid) PNST, which mimicked glandular schwannoma. Symptomatology, imaging characteristics, NGS, and histopathological characteristics that can decipher between different benign PNST subtypes (schwannoma, neurofibroma, glandular, hybrid, etc.), malignant PNSTs (MPNSTs), and TTM are described.

## 1. Introduction

A tumor-to-tumor metastasis (TTM) is a rare metastatic process in which a primary malignant tumor (“donor”) metastasizes to another tumor (“recipient”), most commonly a benign tumor. TTM was first described by Berent in 1902 [1] with the first case being published in 1930 by Fried [2]. The development of multiple primary malignancies in a patient is not uncommon and has been reported to occur in up to as many as 8% of cancer patients [3]. However, TTM is considered very rare, with a 2012 review uncovering only 84 cases in a literature search [4]. Since this 2012 review, there are still less than 200 cases in the literature with only a few reports describing TTMs arising from a thyroid tumor donor with variable recipients including leiomyoma of the uterine corpus, lymphoma arising in the skull bone, clear cell renal cell carcinoma, anaplastic meningioma, and primary pulmonary adenocarcinoma [5–10]. The most common TTM recipient tumors are meningioma and renal cell carcinoma with the most common TTM donor tumors being breast and lung cancer. However, a variety of different donor and recipient tumors have been reported [4].

Herein, to our knowledge, we describe the first case of malignant-to-benign tumor-to-tumor metastasis of well-differentiated PTC into a benign (hybrid) PNST reported in the literature. More specifically, we believe this is the first case described in which a PNST was the recipient tumor of a TTM and therefore the first case in which a metastatic carcinoma deposit mimicked glandular schwannoma ultimately complicating the histopathological diagnosis.

The patient was informed that data concerning this case would be submitted for publication and provided consent.

## 2. Case Presentation

**Figure 1:**
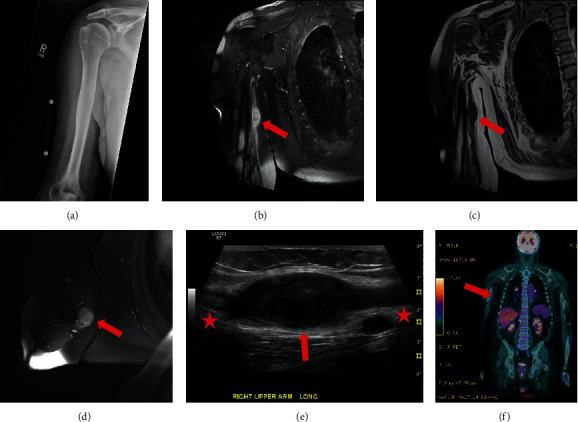
Pre- and postsurgical imaging. (a) A/P shoulder X-ray that was unremarkable. (b) Coronal short tau inversion recovery (STIR) MRI imaging showing an enhancing mass with heterogeneity (red arrow). (c) Precontrast nonfat sat T1-weighted MRI imaging showing the mass to be uniformly hypointense. (d) T1 axial fat-suppression postcontrast MRI showing heterogenous enhancing mass. (e) Image taken during US-guided biopsy showing an enlarged mass (red arrow) arising from nerve (red stars) most consistent for a PNST. (f) Postsurgical PET/CT scan showing some residual FDG signal in the postsurgical bed of the right arm (red arrow) and progression of metastatic PTC (mediastinal adenopathy, right upper paravertebral/subpleural nodule, para-aortic nodule, right medial upper lung nodule, and left upper lobe solid mass).

We present the case of a 74-year-old Caucasian male with a history of PTC, diagnosed in 1999 and treated with total thyroidectomy and radioactive iodine treatment. He did well until he developed dyspnea in 2018, where a CT scan showed multiple pulmonary nodules and lymphadenopathy, which were biopsy-proven PTC. He started a surveillance protocol with image-stable pulmonary nodules over the next four years. In early 2022, he noted a painful mass in the medial aspect of his right arm with radiating pain to his fingers when palpated. These symptoms prompted diagnostic imaging (Figure 1). Right humerus MRI revealed a 3.5 × 2.3 × 1.8 cm solid mass in the neurovascular bundle of the medial arm which was hypointense on T1 and hyperintense on T2 with postcontrast enhancement (Figures 1(b)–1(d), red arrows). He was referred to the orthopaedic oncology clinic for further evaluation, where an ultrasound-guided biopsy was performed (Figure 1(e)). The biopsy findings were nonspecific but suggested a benign lesion. Upon resection of the mass, the ulnar nerve was identified and noted to be grossly enlarged. The mass was identified within the nerve and was carefully dissected away from the central nerve fascicle. Postoperatively, he reported significant improvement in his symptoms with full motor function.

**Figure 2:**
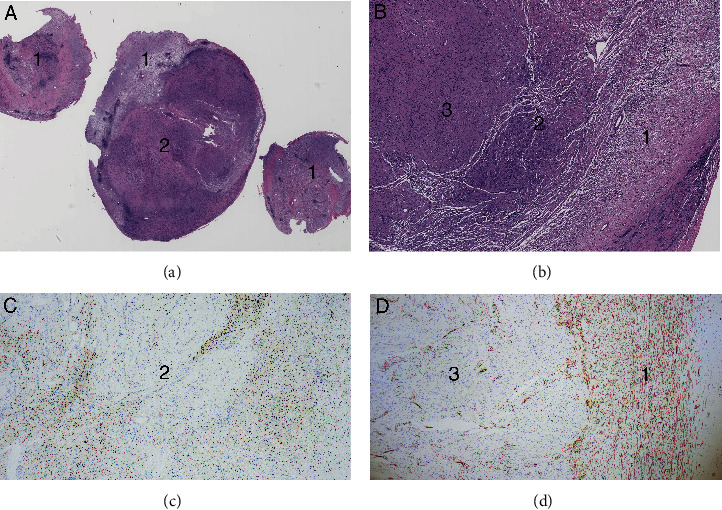
Histopathology of hybrid neurofibroma/schwannoma component. Low-magnification images ((a) 40x and (b) 100x) identify the neurofibroma (A1/B1), schwannoma (B3), and metastatic carcinoma (A2/B2). Immunohistochemistry for SOX10 (c) is positive within the NF and schwannoma portions, but negative in the carcinoma portions (C2). CD34 (d) is negative within the schwannoma component (D3) and exhibits the so called “thumbprint” appearance in the NF component (D1).

Histologic examination showed a benign peripheral nerve sheath tumor (PNST) which exhibited a hybrid of morphologies (Figures 2(a) and 2(b)), with features of a schwannoma as well as a neurofibroma with metastatic PTC deposits. Regions of the neoplasm exhibited loose, myxoid regions with bland spindled cells which were positive for CD34 in a characteristic “thumbprint” pattern of neurofibroma (Figure 2, B1 and D2). Other foci featured an increased cellularity of spindled cells arranged in fascicles and palisades, which were negative for CD34, compatible with schwannoma (Figure 2, B3 and D1). The lesional cells from both components of the PNST were immunoreactive for both S100 and Sox10 (Figure 2(c)).

**Figure 3:**
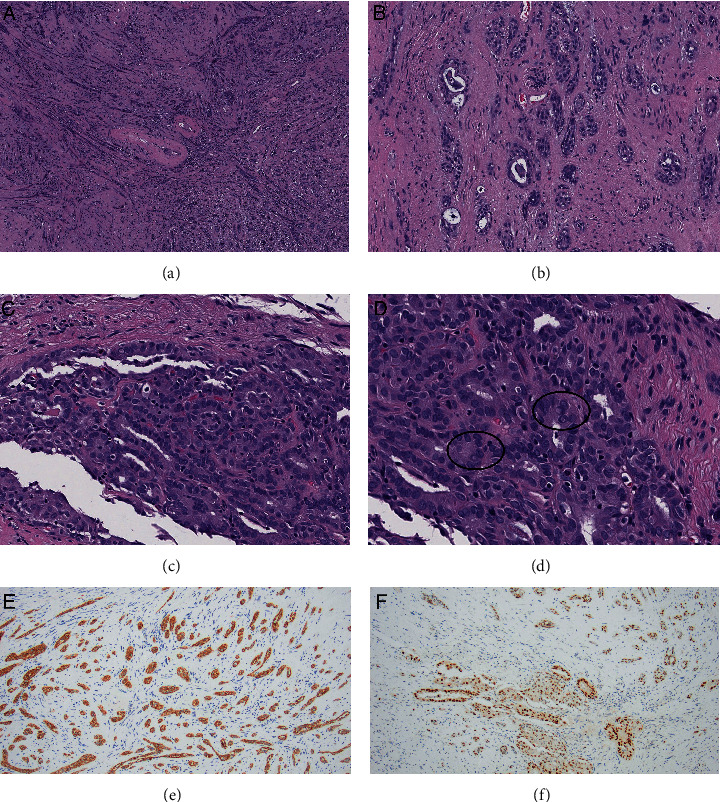
Histopathology of PTC component. Lower magnification images ((a) 40x and (b) 100x) and higher magnification images ((c) 200x and (d) 400x) highlight the metastatic cells arranged in glands. These cells exhibit classical nuclear features of papillary thyroid carcinoma including nuclear grooves and intranuclear pseudoinclusions ((d) circled). Immunohistochemistry for AE1/AE3 (e) and PAX8 (f) are positive within the glands, confirmatory for metastatic PTC.

Centrally located within the PNST were foci of elongated, clustered glandular elements. These glandular cells exhibited atypical morphology with rare cells bearing classical nuclear features of papillary thyroid carcinoma (PTC), including powdery chromatin, nuclear grooves, and prominent intranuclear cytoplasmic inclusions (Figures 3(a)–3(d)). This malignant glandular component was positive for AE1/AE3 (Figure 3(e)) and given the patient's prior history of PTC, and to exclude the possibility of PNST with glandular differentiation, additional immunohistochemical stains were performed. PAX8 (Figure 3(f)) and TTF1 were both positive in the glandular cells, which were negative for Sox10 (Figure 2, C2). Thus, the glandular component was most consistent with a metastatic PTC. The final diagnosis rendered was mixed PNST (4.5 cm) harboring metastatic PTC (1.8 cm) (Figures 2(a) and 2(b)). Surgical resection margins were negative for carcinoma.

A Tempus xT 648 gene next-generation sequencing (NGS) was performed on the tissue, and a Tempus Xf 105 gene NGS cell-free DNA liquid biopsy was performed on peripheral blood. In the tissue, somatic variants were determined in NF2 (c.970del p.Q324fs (NM_000268) frameshift loss-of-function mutation with a variant allele frequency (VAF) of 9.5%), TERT2 (c.-124C>T (NM_198253) promoter mutation variant with a VAF of 8.7%.), and BRAF (c.1799T>A p.V600E (NM_004333) missense gain-of-function variant with a VAF of 6.9%). In the blood, 2 biologically relevant variants in BRAF (c.1799T>A p.V600E NM_004333 missense gain-of-function variant with a VAF of 1.0%) and TP53 (c.722C>T p.S241F (NM_000546) missense loss-of-function variant with a VAF of 0.2%) were found along with a variant of unknown significance in RNF43 (c.443C>G p.A148G (NM_017763) missense variant with a VAF of 47.4%).

Due to these histopathological and NGS findings, a full body PET/CT scan was performed to determine metastatic burden (Figure 1(f)). An area of mild uptake along the medial right upper arm was seen, most likely representing postsurgical changes. Regarding his PTC, there was progression of FDG-avid disease in the lungs/mediastinum when compared to a PET/CT scan performed 5 years prior. Due to the metastasis of his PTC to the PNST and overall progression of his PTC, 14 mg of Lenvima (lenvatinib) daily was prescribed with plans for elevated PET/CT surveillance. Of note, the patient was not tested for a background of NF1 as his circumstance did not meet the clinical diagnostic criteria for neurofibromatosis type 1 or type 2 [11].

## 3. Discussion

The terms “tumor-to-tumor metastasis” and “collision tumor” have often been confused with one another and used incorrectly in the literature. Collision tumors are defined as two neighboring neoplasms, usually from the same organ structure, that invade one another [12], i.e., gastric adenocarcinoma intermixed with gastrointestinal stromal tumor [13]. Tumor-to-tumor metastasis has proved more difficult to define. In 1968, Campbell et al. reviewed the cases of tumor-to-tumor metastasis and made strict criteria for the diagnosis of this uncommon phenomenon which include the following: (1) more than one primary tumor must exist. (2) The recipient tumor must be a true malignant or benign neoplasm. (3) The metastatic neoplasm is a true neoplasm with established growth in the host tumor and not the result of contiguous growth or embolization of tumor cells. (4) The tumors that metastasized to the lymphatic system where lymphoreticular malignant tumors already exist are to be excluded [14]. In the present case, the inclusion of PTC metastasizing to a hybrid PNST meets the above criteria.

Common benign nerve sheath tumors include neurofibromas, schwannomas, and perineuriomas. Schwannomas and perineuriomas are composed of a uniform population of a single cell type (Schwann cells and perineurial cells, respectively), whereas neurofibromas are composed of different cell types, including Schwann cells, fibroblasts, perineurial cells, and scattered axons [15]. PNSTs account for 10-12% of all benign soft tissue tumors. Hybrid PNSTs have only recently been reported in 1998 by Feany et al. [16] where they were described as nerve sheath tumors showing discrete areas of more than one histologic type (hybrid) [17]. Hybrid PNSTs are very rare and are more likely to be in cutaneous regions. Interestingly, our patient's hybrid PNST was deep in the arm.

Peripheral nerve sheath tumors may show heterologous differentiation, including cartilage, bone, and striated muscle [18], with glandular differentiation being the rarest form. Originally described by Garré in 1892 [19], glandular nerve sheath tumors are typically malignant, with few, rare cases of truly benign glandular schwannomas described in the literature [20, 21]. The origin of glandular elements is still debated—early researchers speculated that the glandular elements manifested from heterotopic ependymal tissue, others have hypothesized that the epithelial metaplasia of Schwann cells produces the glandular structures, and some postulate that neural crest cells possess the potential to differentiate into all elements of glandular schwannomas [22, 23]. In our patient, the morphologic and immunophenotypic findings supported a diagnosis of metastatic PTC rather than that of benign glandular differentiation.

An important distinction must be made between PNSTs, a benign entity, and malignant peripheral nerve sheath tumors (MPNSTs). However, MPNSTs are relatively rare, with an incidence ranging from 3 to 10% of soft tissue sarcomas [24]. The differentiation of benign from malignant PNSTs is important for appropriate management plans, and as MPNST is highly malignant with a tendency for recurrence and metastasis, its early diagnosis is necessary. About 50% of MPNSTs occur in the setting of NF1 as these patients have an 8 to 13% risk of developing one in their lifetime. Our patient was never tested for NF1 background as he did not meet the diagnostic clinical criteria for neurofibromatosis type 1 or type 2 [11]. A recent study determined that a smaller size, recognition of contiguity with adjacent nerves, and lesional circumscription favored a benign diagnosis [25]. In contrast, MPNSTs frequently present as larger lesions (>5 cm) with irregular borders and rapid growth by interval imaging or physical exam. Furthermore, MPNSTs are more likely to exhibit divergent differentiation, which can include focal regions of true cartilaginous, myogenic, osseous, angiomatous, and epithelial/glandular differentiation, or any combination thereof [26]. Indeterminant lesions, for which there is uncertainty as to whether they are benign or malignant (as in our case), require subsequent biopsy or surgical removal for accurate histological diagnosis [27]. Unfortunately, biopsy of these tumors may cause severe pain, nerve palsy, or even seeding of malignant tumor cells into visceral organs or other structures [24].

Regarding TTM, characterization and knowledge of tumor biology is important for diagnostic and prognostic information and appropriate patient management. For example, benign PNSTs are usually slow-growing and can remain asymptomatic. If a mass rapidly enlarges or suddenly becomes symptomatic, MPNST and/or TTM should remain on the differential diagnosis. Routine radiological imaging techniques, such as CT and MRI, cannot always reliably exclude the presence of TTM (and has only been studied thoroughly in meningioma donors [4]).

In TTM diagnosis, histology is of utmost importance. The goal should be to define borders of the differing tissue types by cellular morphology and the utilization of immunohistochemistry (especially if the potential metastatic tumor origin is known). Regarding our case, both schwannomas and neurofibromas are diffusely positive for S100 and SOX10 protein. However, in neurofibroma, EMA labels the perineurial cells, and CD34 highlights the stromal cells in a so called “thumbprint” pattern which is beneficial to differentiate from looser (Antoni B) areas of typical schwannoma.

As the glandular component harbored atypical cytology beyond what would be expected for a benign entity, MPNST must be considered in the differential diagnosis of TTM. These tumors typically demonstrate a high mitotic figure rate with variable or absent S100 and/or SOX10 expression, both of which should be strongly positive in schwannomas [15]. Additionally, MPNSTs may also demonstrate divergent differentiation as previously discussed and loss of H3K27 trimethylation [28]. Since our case retained H3K27 staining, MPNST was unlikely, although loss of this marker is not specific. In neural tumors, glandular differentiation may be present in both benign schwannoma and MPNST, both of which were excluded in this case via IHC^18^.

In our case, the glandular component was identified as metastatic carcinoma, aided by prior knowledge of PTC diagnosis and confirmed by the immunophenotype, with diffuse positivity for TTF-1 and PAX8 within these cells [29]. This IHC profile, in conjunction with the morphologic features typically associated with PTC, was diagnostic for metastatic carcinoma of thyroid origin in a background of a benign mixed neural lesion and therefore best classified as TTM.

NGS of the tissue and peripheral blood are novel technologies that can also help in TTM diagnosis and prognosis/treatment of the metastatic tumor. Finding variants that have high specificity and sensitivity for the two (or more) different tumors in the tissue can be suggestive of TTM. In our case, the tissue had somatic variants in BRAF and TERT2 which are commonly mutated in PTC [30] and in NF2 which is commonly mutated in PNSTs [31]. In certain cases, if NGS can be done on the metastatic tumor tissue, peripheral blood, or on a biopsy of the TTM lesion, variant information can provide insights into targeted therapies that may prevent surgical intervention, may aid in surgical intervention by shrinking the lesion, or may treat the TTM if surgery may not be an option due to location and/or patient candidacy.

## 4. Conclusion

This case highlights the possibility that PTC can be the “donor” and that PNSTs can be the “recipient” in TTM cases, albeit a rare event. Furthermore, the PNST in this case was a hybrid PNST showing histological characteristics of both a schwannoma and a neurofibroma, making this case even more unique. This TTM case was diagnostically challenging due to an equivocal biopsy finding, symptomatology consistent with a potential MPNST, and final histopathology mimicking a glandular schwannoma. Ultimately, TTM deposits could be the first metastatic event or indicative of the extent of a patient's metastatic disease (as in this case) and therefore, vigilance is recommended.

## Data Availability

Information and data can be accessed from the submitting author (juan.m.colazo@vanderbilt.edu) or the corresponding author (ginger.e.holt@vumc.org) via email.
